# Safety of the Recombinant Cholera Toxin B Subunit, Killed Whole-Cell (rBS-WC) Oral Cholera Vaccine in Pregnancy

**DOI:** 10.1371/journal.pntd.0001743

**Published:** 2012-07-24

**Authors:** Ramadhan Hashim, Ahmed M. Khatib, Godwin Enwere, Jin Kyung Park, Rita Reyburn, Mohammad Ali, Na Yoon Chang, Deok Ryun Kim, Benedikt Ley, Kamala Thriemer, Anna Lena Lopez, John D. Clemens, Jacqueline L. Deen, Sunheang Shin, Christian Schaetti, Raymond Hutubessy, Maria Teresa Aguado, Marie Paule Kieny, David Sack, Stephen Obaro, Attiye J. Shaame, Said M. Ali, Abdul A. Saleh, Lorenz von Seidlein, Mohamed S. Jiddawi

**Affiliations:** 1 International Vaccine Institute, Seoul, Korea; 2 Ministry of Health and Social Welfare, Stonetown, Zanzibar; 3 World Health Organization, Geneva, Switzerland; 4 Biocenter, University of Vienna, Vienna, Austria; 5 Swiss Tropical and Public Health Institute, Basel, Switzerland; 6 University of Basel, Basel, Switzerland; 7 Johns Hopkins School of Public Health, Baltimore, Maryland, United States of America; 8 Michigan State University, East Lansing, Michigan, United States of America; 9 Public Health Laboratory Ivo de Carneri, Chake-Chake, Zanzibar; 10 Menzies School of Health Research, Casuarina, Northern Territory, Australia; Massachusetts General Hospital, United States of America

## Abstract

**Introduction:**

Mass vaccinations are a main strategy in the deployment of oral cholera vaccines. Campaigns avoid giving vaccine to pregnant women because of the absence of safety data of the killed whole-cell oral cholera (rBS-WC) vaccine. Balancing this concern is the known higher risk of cholera and of complications of pregnancy should cholera occur in these women, as well as the lack of expected adverse events from a killed oral bacterial vaccine.

**Methodology/Principal Findings:**

From January to February 2009, a mass rBS-WC vaccination campaign of persons over two years of age was conducted in an urban and a rural area (population 51,151) in Zanzibar. Pregnant women were advised not to participate in the campaign. More than nine months after the last dose of the vaccine was administered, we visited all women between 15 and 50 years of age living in the study area. The outcome of pregnancies that were inadvertently exposed to at least one oral cholera vaccine dose and those that were not exposed was evaluated. 13,736 (94%) of the target women in the study site were interviewed. 1,151 (79%) of the 1,453 deliveries in 2009 occurred during the period when foetal exposure to the vaccine could have occurred. 955 (83%) out of these 1,151 mothers had not been vaccinated; the remaining 196 (17%) mothers had received at least one dose of the oral cholera vaccine. There were no statistically significant differences in the odds ratios for birth outcomes among the exposed and unexposed pregnancies.

**Conclusions/Significance:**

We found no statistically significant evidence of a harmful effect of gestational exposure to the rBS-WC vaccine. These findings, along with the absence of a rational basis for expecting a risk from this killed oral bacterial vaccine, are reassuring but the study had insufficient power to detect infrequent events.

**Trial Registration:**

ClinicalTrials.gov NCT00709410

## Author Summary

Pregnant women are more vulnerable to complications of cholera than other people. It would be helpful to include pregnant women in vaccination campaigns against cholera but pregnant women and their unborn children are highly vulnerable to the potential adverse effects of biological products such as vaccines. The safety of oral cholera vaccines in pregnant women has up to now not been evaluated. During a large mass cholera vaccination campaign in Zanzibar in 2009, women were advised not to participate if they thought they may be pregnant. The large majority (955 or 83%) of women residing in the study area who were to be pregnant during the 9 months following the vaccinations did not participate in the campaign. The remaining 196 (17%) women received the vaccine. A comparison between vaccine exposed and unexposed pregnancies did not reveal any significant differences in outcome between the two groups. The small number of miscarriages, infant deaths and ill infants was similarly distributed between the two groups. These findings are reassuring but continued monitoring of this vaccine when given during pregnancy is recommended.

## Introduction

The recombinant cholera toxin B subunit, killed whole-cell oral cholera (rBS-WC, Dukoral) vaccine, has been found to be safe and protective in a range of settings over the last 30 years [1], [2], [3]. This vaccine is mainly used by tourists visiting endemic areas [4] where the control of cholera has traditionally been based on safe water supply, sanitation and health education [5]. A more affordable oral cholera vaccine which could be used more widely in endemic settings has recently been developed, licensed, and prequalified for purchase by UN agencies [6]. This second generation killed oral cholera vaccine (Shanchol) is composed of a different set of *V.cholerae* strains than the rBS-WC vaccine, includes not only O1 but also an O139 strain, does not include the recombinant B subunit (rBS), therefore does not require buffer for administration, and has afforded 66% protection during a 3 year trial in Kolkata, India [7]. In early 2010, the Strategic Advisory group of the World Health Organization (WHO) recommended that oral cholera vaccines be used preventively as well as reactively in the management of cholera outbreaks [8].

Since cholera tends to affect all age groups in endemic settings and during outbreaks, mass vaccination is considered an important vaccine deployment strategy. To achieve maximum impact of mass cholera vaccination, it is crucial to immunize the highest possible percentage of the population at risk. This includes women in the reproductive age group, defined here as being between 15 and 50 years old. In endemic and epidemic settings, women are at high risk for cholera and other diarrhoeal diseases, not least because mothers tend to be exposed to infectious children [9]. Without prompt rehydration, cholera during pregnancy can result in abortions, premature childbirth and maternal death [10], [11]. There are good reasons for women in the reproductive age group in endemic areas to participate in interventions that prevent cholera. Excluding potentially pregnant women from mass vaccination campaigns is logistically and ethically challenging. But administering oral cholera vaccines to this highly vulnerable population causes a dilemma since the safety of the vaccine during pregnancy has not been documented. There are several reasons why it is thought that oral cholera vaccines are unlikely to have a harmful effect on foetal development. First, the bacteria in the rBS-WC vaccine are killed and do not replicate. Second, the vaccine antigens act locally on the gastro-intestinal mucosa is not absorbed and does not enter the maternal or foetal circulation. Third rBS-WC vaccines don't trigger systemic reactions (e.g. fever) linked to abortions early in pregnancy. However, no actual safety studies of the rBS-WC vaccine in pregnancy have been carried out [12].

The uncertainty regarding the use of the vaccine during pregnancy has resulted in differing recommendations. The recommendations from the WHO state the following. “The primary targets for cholera vaccination in many endemic areas are preschool-aged and school-aged children. Other groups that are especially vulnerable to severe disease and for which the vaccines are not contraindicated may also be targeted, such as pregnant women and HIV-infected individuals.” [13]. The package insert of Dukoral, states: “The effect of DUKORAL [Oral, Inactivated Travellers' Diarrhoea and Cholera Vaccine] on embryo-foetal development has not been assessed and animal studies on reproductive toxicity have not been conducted. No specific clinical studies have been performed to address this issue. The vaccine is therefore not recommended for use in pregnancy. However, DUKORAL is an inactivated vaccine that does not replicate. DUKORAL is also given orally and acts locally in the intestine. Therefore, in theory, DUKORAL should not pose any risk to the human foetus. Administration of DUKORAL to pregnant women may be considered after careful evaluation ofthe benefits and risks.” The package insert of the second generation vaccine (Shanchol ) uses similarly guarded language: “The vaccine is not recommended for use in pregnancy. However, Shanchol is a killed vaccine that does not replicate, is given orally and acts locally in the intestine. Therefore, in theory, Shanchol should not pose any risk to the human foetus. Administration of Shanchol to pregnant women may be considered after careful evaluation of the benefits and risks in case of a medical emergency or an epidemic.”

A mass oral cholera vaccination was conducted in Zanzibar in 2009. Pregnant women were advised not to participate in the campaign. To assess whether any pregnant women had inadvertently received the vaccine, and to investigate birth outcomes, we visited all women residing in the study area and in the reproductive age group more than nine months after the last dose of the vaccine had been administered. The objective of the study was to determine whether there was any difference between the outcomes of pregnancies exposed and not exposed to the oral rBS-WC cholera vaccine.

## Methods

The study methods have been described in more detail in the accompanying paper estimating the effectiveness of the vaccine [14].

### Ethics statement

The study was conducted according to the principles expressed in the Declaration of Helsinki. Individual verbal consent was obtained from each respondent after the purpose of the study was explained. The Institutional Review Board of the Government of Zanzibar (ZAMREC), of the International Vaccine Institute, Seoul, Korea, and the Research Ethics Review Committee of the World Health Organization, Geneva, Switzerland approved this project.

The informed consent process was done in several phases. Community informed consent was obtained through meetings with the local leaders (She has). A multistage community outreach campaign was conducted to disseminate information about the planned study activities. During the census, individual verbal informed consent was obtained prior to the interview of each household head or his or her representative. During the mass vaccination, individual verbal informed consent was obtained from each participant or from his or her guardian, if they were less than 18 years of age. In addition, verbal assent from children 12 to 17 years of age was obtained. The participants received information regarding the vaccine, including advice for children less than 2 years of age and pregnant women not to receive the vaccine. There was no screening for pregnancy prior to vaccine administration.

The interview of pregnant women was closely linked with the census, for which oral consent was provided. Like the census interview, the interview of pregnant women posed minimal risks and oral consent was deemed appropriate. Provision of oral consent by each participant was documented in a logbook. The use of oral consent was approved by the ethics review boards. After the surveillance was completed the three ethics review boards were informed about the conduct and the findings of the birth surveillance.

### Study site

The archipelago of Zanzibar lies about 50 kilometres east of mainland Tanzania and consists of two main islands, Unguja and Pemba, as well as smaller islets. Zanzibar had a population of about 1.1 million in 2009. In Unguja, we included the *shehias* of Chumbuni, Karakana, and Mtopepo, which are informal, urbanized areas extending from the capital, Zanzibar City also known as Stonetown. These shehias arose without the corresponding development of adequate water and sanitation facilities. In Pemba, we included the *shehias* of Mwambe, Kengeja, and Shamiani, located in the mainly rural southeast of the island.

### Vaccine

Each dose of the rBS-WC cholera vaccine (Dukoral ™, SBL Vaccine AB, Sweden) consists of ca. 1×10^11^ vibrios [12]:

- *Vibrio cholerae* O1 Inaba classical strain, heat inactivated (ca. 2.5×10^10^ vibrios)
- *V. cholerae* O1 Inaba El Tor strain, formalin inactivated (ca. 2.5×10^10^ vibrios)
- *V. cholerae* O1 Ogawa classical strain, heat inactivated (ca. 2.5×10^10^ vibrios)
- *V. cholerae* O1 Ogawa classical strain, formalin inactivated (ca. 2.5×10^10^ vibrios)
- Recombinant cholera toxin B subunit (1 mg)

The full dose of vaccine was mixed with 75 or 150 ml of buffer solution for participants aged from two to six years and over six years, respectively.

### Census

A formal census was conducted from November to December 2008, collecting demographic and socio-economic information. Verbal informed consent was obtained from the head of each household prior to the interviews. The number of household members, ownership of various capital goods and household building materials were recorded. Data was directly entered into handheld computers, also known as personal digital assistants (PDA) [15]. A unique identification number was assigned to each resident in the study sites. After the census was completed, household identification cards were distributed in early January 2009. At the time of card distribution, all healthy, non-pregnant residents of the study sites who were two years of age and older were invited to participate in the mass vaccination campaign. Study residents were requested to bring their household identification cards when coming to a vaccination outpost to facilitate identification. In August 2009, a second census was conducted in the study sites to update the study population database.

### Mass vaccination campaign

The mass vaccination campaign was implemented by the Expanded Program on Immunization of the Zanzibar Ministry of Health and Social Welfare with WHO technical support. The first round of immunizations was conducted from January 11 to 26, 2009, the second round from February 7 to 16, 2009. The vaccine vial was shaken, opened and its contents poured into a cup with buffer solution and stirred. The participants drank the mixture under direct observation and completeness of ingestion was recorded. During the first round, a card was issued to each vaccine recipient to record the subject's name, age, address, household head, date of vaccination, and completeness of ingestion of the dose. At the time of dosing, this information was also recorded in a PDA-based vaccination registry. Only those who had received a first dose (as documented in the vaccination card or the PDA registry) were given a second dose of the vaccine.

### Birth surveillance

The birth surveillance was conducted more than 9 months after the mass vaccination campaign was completed, between January 15 and February 15, 2010. A list of all women between 15 and 50 years of age at the time of the vaccination campaign and living in the study area was prepared based on the study population database. Following training in study procedures fieldworkers visited the listed women and asked whether they had been pregnant in 2009. Women who had been pregnant were asked about the following: the date of delivery, duration and outcome of the pregnancy based on their last menstrual period, number of deliveries, age of the last child born before this delivery, antenatal clinic attendance during this pregnancy and person who attended the delivery. Birth outcomes were described as miscarriage or live births. We further defined a miscarriage as either a spontaneous abortion or a stillbirth. A spontaneous abortion was defined as a termination of a pregnancy within 20 weeks of conception. A stillbirth was defined as a foetus born after 20 weeks of gestation without a pulse. Live births that died later during infancy were described as infant deaths. For live births, the disposition of the baby was recorded. During the visit the field worker asked whether the baby is free from recurring illness, without gross malformations, and is feeding, urinating, defecating, crying, sleeping and growing normally. For the purpose of this surveillance, a recurrent illness was defined as an illness lasting more than two weeks or occurring twice or more often [16]. Only illnesses requiring the attention of medical staff were included. A gross malformation was defined as a physical defect present in a baby at birth. It includes any abnormality visible on a naked baby (e.g. cleft lip or palate, Down syndrome, spina bifida, limb defects, etc.). Whether feeding, urinating, defecating, crying, sleeping and growing was within the normal range was recorded according to the mother's definition. If the field worker considered the infant as sick or abnormal, the infant was seen by a paediatrician. The paediatrician completed a standardized history, physical examination and assessment and provided treatment or referral according to national guidelines. The field workers and paediatricians were blinded regarding the vaccination status of the mother.

### Analysis

The information collected during birth surveillance was linked to the population census and vaccination databases. Receipt of the cholera vaccine during the mass immunization program was ascertained based on the vaccination database. Linkage to the vaccination registry was made blinded to pregnancy outcome. Baseline data on socio-behavioural, economic, and environmental variables were obtained from the census database.

To calculate the date of conception, we subtracted the duration of the pregnancy (as defined by the mother in weeks based on the last menstrual period) from the date of delivery. The pregnancy was considered exposed to the vaccine if the period from conception to delivery included the dates when the woman received at least one vaccine dose. Additionally, because it is difficult to know the exact date of conception, we included pregnancies with calculated conception dates within two weeks before ingestion of the first vaccine dose as potentially exposed. A pregnancy was considered unexposed if the period from two weeks before the calculated conception date to the date of delivery did not include receipt of any oral cholera vaccine dose. We compared the frequency of adverse birth outcomes between exposed and unexposed pregnancies.

The number of miscarriages, live infants and infant deaths (birth outcomes) among the exposed and unexposed pregnancies were initially compared using chi-square or Fisher's exact test, as appropriate. Characteristics of women who had exposed and unexposed pregnancies were compared using chi-square and Student's t-test for binary/categorical and continuous variables, respectively. In the assessment of the risk for negative outcomes (miscarriage and infant sickness, abnormality or death), a stepwise elimination method was used to select variables most closely associated with exposure and non-exposure and to fit them into a logistic regression model. All p values and 95% confidence intervals were interpreted in a two-tailed fashion. Statistical significance was designated as a p value less than 0.05. Stata/SE 8 (Stata Corporation, Texas, USA) was used for statistical analysis.

## Results

The population census enumerated 14,564 women between 15 and 50 years of age residing in the study sites. During the birth surveillance, 13,736 (94%) of this population were located and interviewed. Women who participated had a significantly different health care utilization pattern, tended to be from a lower socio-economic background as suggested by the possession of fewer capital items (mobile phone, bicycle etc.), came from larger households and tended to be less well educated (Table S1).

**Figure 1:**
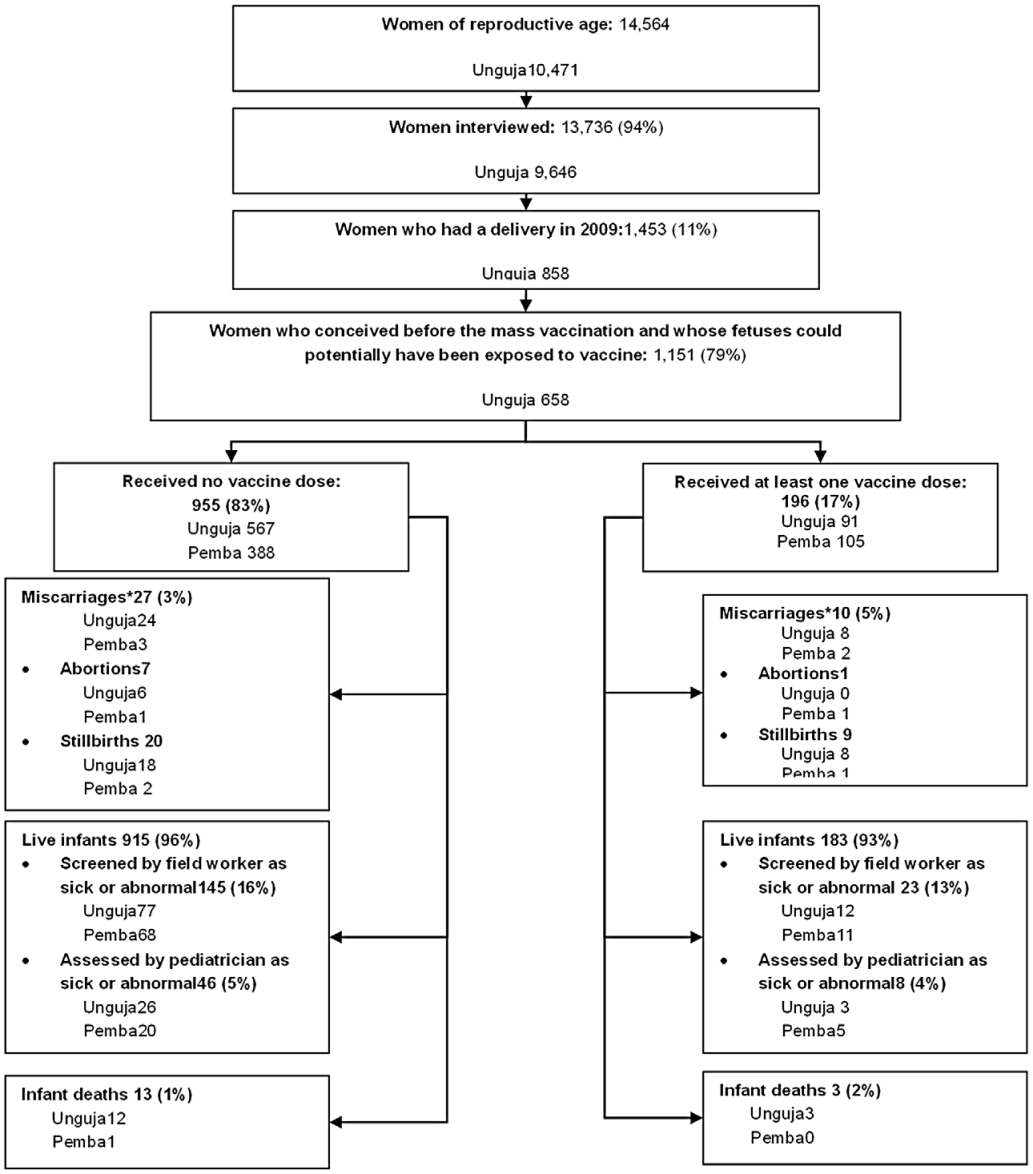
Flow of study participants. *A miscarriage was defined as either an abortion or a stillbirth. An abortion was defined as a termination of a pregnancy within 20 weeks of conception. A stillbirth was defined as a fetus delivered after 20 weeks of gestation without a pulse.

Out of the interviewed women, 1,453 (11%) had a delivery in 2009; and 1,151 (79%) of these deliveries occurred during the period where the foetus could have been exposed to the vaccine. The large majority 955 (83%) out of these 1,151 mothers had not been vaccinated; the remaining 196 (17%) mothers had received at least one dose of the oral cholera vaccine (82 received 1 dose, 114 received 2 doses). The flow of the pregnant women is shown in Figure 1.

We compared the outcomes of pregnancies exposed and unexposed to the cholera vaccine (Table 1). There was no statistically significant difference in the number of miscarriages among the exposed compared to the unexposed pregnancies [10/196 (5%) vs. 27/955 (3%), adjusted odds ratio (AOR) 1.62 (95% confidence interval (95% CI 0.76 to 3.43)]. Similarly, there was no statistically significant difference in the number of infant deaths among the exposed compared to the unexposed non-miscarriage pregnancies [3/186(2%) vs. 13/928 (1%), AOR 1.46 (95% CI 0.41 to 5.29)]. The frequency of infant illness and abnormalities among the live infants verified by a paediatrician was 8/183 (4%) among the exposed versus 46/915 (5%) among the unexposed (AOR 0.79, 95^th^ CI 0.36 to 1.75). Logistic regression models, adjusted for variation in background characteristics, found no significant difference in the frequency of miscarriages, sickness or abnormality, and infant deaths between the exposed and unexposed pregnancies.

**Table 1:** Adjusted odds ratio of exposure to vaccine for negative outcomes using logistic regression models.

	Exposed (n = 196) N (%)	Unexposed (n = 955) N (%)	P value un-adjusted	Adjusted odds ratio (95% CI)	P value adjusted
Miscarriages	10 (5.1)	27 (2.8)	0.10	1.62^1^	0.21
Live births	186 (94.9)	928 (97.2)		(0.76–3.43)	
Deaths	3 (1.6)	13 (1.4)	0.82	1.46^2^	0.56
Live infants	183 (98.4)	915 (98.6)		(0.41–5.29)	
Sick based on paediatrician's examination	8 (4.4)	46 (5.0)	0.70	0.79^3^	0.56
Healthy infants	175 (95.6)	869 (95.0)		(0.36–1.75)	

1 Adjusted for: motorcycle ownership and number of deliveries.

2 Adjusted for: household construction materials and sex of the baby.

3 Adjusted for: household size and travel.

**Figure 2:**
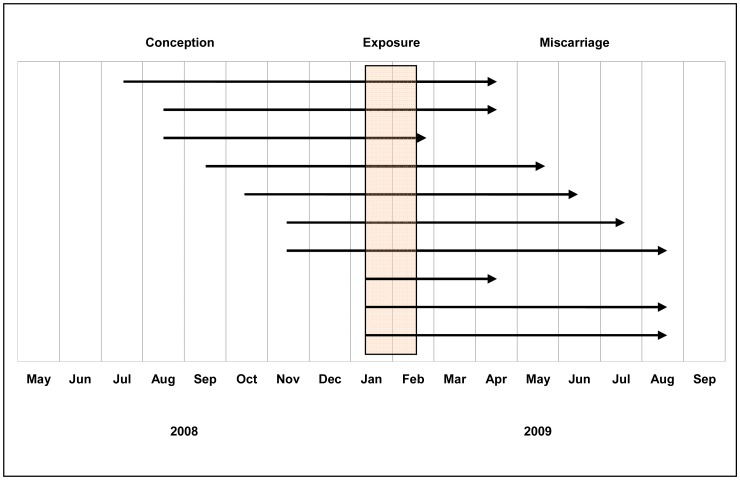
Timing of vaccine exposure during pregnancy of the 10 miscarriages.

We assessed the timing of the exposure to the cholera vaccine in relation to the gestational period of the ten miscarriages (Figure 2). Vaccine exposure occurred during the first trimester in three, during the second trimester in four, and during the third trimester in three pregnancies.

We compared individual and household characteristics of the mothers who had exposed and unexposed pregnancies (Table 2). Pregnant women who participated in the mass vaccination campaign differed in several aspects from pregnant women who didn't participate in the vaccinations. The women who received the vaccine were significantly older, had had more deliveries, attended antenatal care less frequently, had more frequently lived in the same household during the past 5 years and lived in a larger household with lower socio-economic status as suggested by the ownership of capital items and household construction materials.

**Table 2:** Comparison of individual and household characteristics between mothers exposed and unexposed to the oral cholera vaccine.

	Exposed	Unexposed	P value*
	n = 196	n = 955	
Mean age (SD)	30.1(7.9)	28.8(7.2)	0.04
Mean no of deliveries (SD)	5.2(2.8)	4.2(2.8)	<.01
No (%) with educational level as follows:			
Illiterate	55(28.1)	235(24.7)	0.33
Completed primary level and above	141(71.9)	716(75.3)	
No (%) who attended antenatal clinic during this pregnancy	191(97.5)	949(99.4)	0.03
No (%) whose delivery was attended by a health professional (nurse, clinical officer or doctor)	3(1.5)	13(1.4)	0.85
Mean age of her last child born before this delivery (SD)	3.3(1.9)	3.5(1.8)	0.06
No (%) with twins born during this delivery	6(3.1)	58 (6.1)	0.09
No (%) with household role as follows:			
Head of household	10(5.1)	43(4.5)	0.97
Daughter of household head	14(7.1)	63(6.6)	
Wife of household head	158(80.6)	776(81.3)	
Other	14(7.1)	73(7.6)	
No (%) residing during the past 5 years:			
In the same household	155(79.1)	668(70.0)	0.01
In another household	41(20.9)	286(30.0)	
Mean no of household members (SD)	6.6(2.9)	5.9(2.9)	<.01
No (%) who own or with household members who own a:			
Mobile phone	65(33.3)	373(40.6)	0.06
Bicycle	74(37.8)	403(43.7)	0.13
Motorcycle or scooter	11(5.6)	65(7.0)	0.47
Car or truck	1(0.5)	11(1.2)	0.40
No (%) whose household has:			
Electricity	52(26.5)	310(33.7)	0.05
A radio	132(67.3)	636(68.9)	0.67
A television set	40(20.4)	223(24.2)	0.26
A refrigerator	15(7.7)	117(12.7)	0.05
A cemented, tiled or carpeted floor (versus mud)	118(60.2)	624(67.5)	0.05
A cemented or tiled wall (versus thatched)	140(71.4)	722(78.1)	0.04
A metal or tiled roof (versus thatched)	172(87.8)	813(88.0)	0.93
A safe main source of drinking water**	130(66.3)	664(71.9)	0.12

*: The p-values were derived by comparing the differences between the two groups (t-test for mean comparison for continuous variables and chi-square test for binary/categorical variables).

**: Safe water sources included: protected well, tap water and bottled water; unsafe water sources included: unprotected well, pond, river/stream, spring, and other.

## Discussion

This is the first report of the safety of the rBS-WC oral cholera vaccine administered during pregnancy. We found no significant differences in birth outcomes among pregnancies exposed and unexposed to the rBS-WC oral cholera vaccine. Among the 196 pregnancies with gestational exposure to the vaccine, there was no evidence of a statistically significant increase in the number of foetal losses or infant deaths compared to unexposed pregnancies. There was a slightly higher percentage of miscarriages in pregnancies exposed to the oral cholera vaccine than in pregnancies not exposed. This trend did not reach statistical significance and is likely explained by chance.

The study has several limitations. First, foetal losses were probably under-reported since pregnancy is often denied until late into gestation for complex cultural reasons. Second, and more importantly, exposure or non-exposure to the vaccine was not randomized. Instead prior to vaccination women were advised not to participate in the vaccinations if they were pregnant and there was no mandatory pregnancy testing prior to vaccination. This element of self-selection may have led to bias. Third, an element of recall bias can't be ruled out, namely women may have recalled adverse outcomes more frequently when they had been vaccinated than unvaccinated women. Fourth, the study detected 196 pregnancies. Even though the study is the largest to date, the overall number of pregnancies (196) is small and has limited power to detect infrequent adverse events. Fifth our sampling method does not detect maternal deaths. Finally, 6% of the eligible women did not participate in this birth surveillance study. Considering that the large majority (94%) of eligible women participated in the study it seems unlikely that this finding has introduced bias.

To help ensure the validity of the results, we performed the following procedures: To ensure the complete detection of all pregnancies in the study site, we visited and interviewed all women in reproductive age enumerated in the census. Extensive information about potentially confounding variables was available since data on baseline characteristics of individuals and households were collected during the census and during the interview of the mothers, which were controlled for in the analyses. Birth outcomes were linked in a blinded fashion to vaccination status in the database in order to avoid potential observer bias.

Pregnant women who participated in the mass vaccination campaign were older, had had more deliveries, came from bigger households and had lived in the same household for a longer period than pregnant women who did not participate. Younger pregnant women from smaller, better-off households may well perceive themselves at a lower risk for gastro-enteric infections than more experienced, older women from a lower socio economic background. Similar observations of an inverse relationship between participation in free mass vaccination campaigns and socio-economic status have been reported from Kolkata, India [17] and Hue, Vietnam [18]. Alternatively younger women from a higher socio-economic background have a better understanding of the potential risks of vaccination during pregnancy than women from a lower socio-economic background.

### Conclusions

This study found no significant increase in adverse events involving the foetus or newborn among pregnant women who inadvertently received killed oral cholera vaccine. Because the sample size was small, our findings cannot rule out the possibility that rBS-WC vaccine could cause adverse events during pregnancy, but the study provides reassurance that such events are not common. The findings from this study support the current recommendation that killed oral cholera vaccine is not contraindicated during pregnancy, but the decision to administer the vaccine should depend on the epidemiological context and after weighing the potential benefits and risks [12]. Randomized, controlled studies of the rBS-WC vaccine in pregnant women are ethically not justifiable, but future, larger mass vaccinations may allow further evaluation of birth outcomes after inadvertent exposure of pregnant women to the vaccine.

## Supporting Information

Table S1 **Baseline characteristics of women who participated and didn't participate in the birth surveillance.**
(DOC)
