# Supplementary material for: Safety of the Recombinant Cholera Toxin B Subunit, Killed Whole-Cell (rBS-WC) Oral Cholera Vaccine in Pregnancy
Source: PLoS Negl Trop Dis. 2012 Jul 24;6(7):e1743. doi: 10.1371/journal.pntd.0001743 (PMC3404114; doi:10.1371/journal.pntd.0001743)
Supplement: Table S1 — Baseline characteristics of women who participated and didn't participate in the birth surveillance. (DOC) [file pntd.0001743.s001.doc]

**Table S1: Baseline characteristics of women who participated and didn’t participate in the birth surveillance.**

| **Risk factor** | **Participated**  **n = 13,736 (%)** | **Not participated**  **n = 828**  **(%)** | **P-Value** |
| --- | --- | --- | --- |
| Health seeking behavior |  | | |
| Stay home | 2902(21.22) | 139(17.01) | 0.002 |
| Pharmacy | 1010(7.39) | 45(5.51) |
| Hospital | 9735(71.18) | 632(77.36) |
| Others | 29(0.21) | 1(0.12) |
| Relationship to the head |  | | |
| Head | 767(5.58) | 83(10.02) | <0.001 |
| Daughter | 3912(28.48) | 152(18.36) |
| Wife | 6567(47.81) | 433(52.29) |
| Others | 2490(18.13) | 160(19.32) |
| Household size mean(SD) | 6.9(3.27) | 5.7(2.82) | <0.001 |
| Bicycle |  | | |
| Yes | 6051(44.76) | 332(40.15) | 0.009 |
| No | 7467(55.24) | 495(59.85) |
| motorcycle/scooter |  | | |
| Yes | 1547(11.46) | 108(13.06) | 0.163 |
| No | 11952(88.54) | 719(86.94) |
| Car/truck |  | | |
| Yes | 214(1.59) | 18(2.18) | 0.196 |
| No | 13234(98.41) | 809(97.82) |
| Electricity |  | | |
| Yes | 5702(42.23) | 430(52) | <0.001 |
| No | 7801(57.77) | 397(48) |
| Radio |  | | |
| Yes | 9404(69.59) | 559(67.68) | 0.246 |
| No | 4109(30.41) | 267(32.32) |
| TV |  | | |
| Yes | 4202(31.18) | 283(34.3) | 0.061 |
| No | 9273(68.82) | 542(65.7) |
| Refrigerator |  | | |
| Yes | 2565(19.04) | 162(19.59) | 0.699 |
| No | 10904(80.96) | 665(80.41) |
| Mobile |  | | |
| Yes | 6036(44.99) | 415(50.8) | 0.001 |
| No | 7379(55.01) | 402(49.2) |
| Floor |  | | |
| cement/Tile/Carpet | 10056(74.35) | 694(83.82) | <0.001 |
| Other | 3470(25.65) | 134(16.18) |
| Wall |  | | |
| Cement/Tile | 11224(82.98) | 806(97.34) | <0.001 |
| Others | 2302(17.02) | 22(2.66) |
| Education level |  | | |
| Illiterate | 2423(17.69) | 90(10.98) | <0.001 |
| Primary + | 11274(82.31) | 730(89.02) |
| Water source |  | | |
| Safe | 10701(79.11) | 744(89.86) | <0.001 |
| Unsafe | 2825(20.89) | 84(10.14) |
| Residence within 5 years |  | | |
| Same household | 10507(76.52) | 457(55.33) | <0.001 |
| Other | 3224(23.48) | 369(44.67) |
